# BMI mediates the association between low educational level and higher blood pressure during pregnancy in Japan

**DOI:** 10.1186/1471-2458-13-389

**Published:** 2013-04-25

**Authors:** Seung Chik Jwa, Takeo Fujiwara, Akira Hata, Naoko Arata, Haruhiko Sago, Yukihiro Ohya

**Affiliations:** 1 Department of Social Medicine, National Research Institute for Child Health and Development, National Center for Child Health and Development, 2-10-1, Setagaya-ku, Tokyo, Japan; 2 Department of Public Health, Chiba University Graduate School of Medicine, Chiba, Japan; 3 Department of Women’s Health, National Center for Child Health and Development, Tokyo, Japan; 4 Center for Maternal-Fetal and Neonatal Medicine, National Center for Child Health and Development, Tokyo, Japan; 5 Department of Medical Specialties, National Center for Child Health and Development, Tokyo, Japan

**Keywords:** Socioeconomic status, Pregnancy, Blood pressure, Educational level, Pregnancy-induced hypertension

## Abstract

**Background:**

Research investigating the association between socioeconomic status (SES) and blood pressure (BP) during pregnancy is limited and its underlying pathway is unknown. The aim of this study was to investigate the mediators of the association between educational level as an indicator of the SES and BP in early and mid-pregnancy among Japanese women.

**Methods:**

Nine hundred and twenty-three pregnant women in whom BP was measured before 16 weeks and at 20 weeks of gestation were enrolled in this study. Maternal educational levels were categorized into three groups: high (university or higher), mid (junior college), and low (junior high school, high school, or vocational training school).

**Results:**

The low educational group had higher systolic (low vs. high, difference = 2.39 mmHg, 95% confidence interval [CI]: 0.59 to 4.19) and diastolic BP levels (low vs. high, difference = 0.74 mmHg, 95% CI: –0.52 to 1.99) in early pregnancy. However, the same associations were not found after adjustment for pre-pregnancy body mass index (BMI). BP reduction was observed in mid-pregnancy in all three educational groups and there was no association between educational level and pregnancy-induced hypertension.

**Conclusion:**

In Japanese women, the low educational group showed higher BP during pregnancy than the mid or high educational groups. Pre-pregnancy BMI mediates the association between educational level and BP.

## Background

Blood pressure (BP) control during pregnancy is crucial for the safety of both mothers and neonates. In previous reports, BP values during pregnancy have been shown to be associated with a continuous inverse effect in fetal growth. Systematic sampling with 24-h ambulatory BP monitoring during pregnancy indicated that a 5-mmHg increase in the mean diastolic BP was inversely associated with a 68-g decrease in birth weight in normotensive [1], and a 68.5-g decrease in hypertensive pregnancies [2]. In addition, subsequent BP elevation results in occurrence of maternal pregnancy-induced hypertension (PIH). High BP in early and mid-pregnancy is strongly associated with the later occurrence of PIH [3,4], which affects 3–10% of all pregnancies and is associated with high levels of maternal, fetal, and neonatal morbidity and mortality [5]. The long-term prognosis of women with a history of PIH also includes increased risk of future cerebrovascular disease, cardiovascular disease, and renal disease [6,7].

A low socioeconomic status (SES) is reported as a risk factor for high BP in adult people [8-10]. This inverse gradient is stronger and more consistent in women than in men. Although it is well known that pregnancy is regarded as a stress test for future hypertension [11,12], few studies have investigated the association between SES and BP during pregnancy [13]. Furthermore, mediators of the association between SES and BP are not well known. Silva et al. reported that the association between educational levels and BP was mediated by pre-pregnancy BMI; however, weight gain, smoking, alcohol intake, or salt intake might be other mediators for this link as these factors were identified in other non-pregnant adult studies [14-17]. In addition, previous literature on SES and BP during pregnancy is limited to Western societies. Japanese diet, lifestyle, or health system might affect on the association between SES and BP in a different manner [18,19]. Thus, the purpose of this study is to investigate the association between educational levels as an indicator of SES and BP in early and mid-pregnancy, PIH, and to elucidate the mediating factors of these associations among Japanese pregnant women.

## Methods

### Study population

This study was a part of the Tokyo-Children’s Health, Illness, and Development study (T-CHILD Study), a single-center, prospective, birth cohort study conducted at the National Center for Child Health and Development (Tokyo, Japan). Study participants were enrolled before 16 weeks of gestation at the obstetrical department from October 2003 to December 2005. The institutional review board at the National Center for Child Health and Development approved this study (Institutional review board approval no. 52 & 341). Written informed consent was obtained from all participants. This study was a secondary analysis of the data.

The inclusion criteria of the current study were access to educational information, BP measurement before 16 weeks of gestation (early pregnancy) and at 20 weeks of gestation (mid-pregnancy), and delivery at the National Center for Child Health and Development after 22 weeks of gestation. We excluded women with multiple pregnancies. In total, 923 participants were enrolled in this study.

### Educational level

Information on the educational level was obtained from a questionnaire at the time of enrollment. Participants were divided into 3 educational groups: high (university or higher), mid (junior college), and low (less than high school, high school or vocational training school). We categorized the participants with vocational school education into the low educational group because vocational schools usually do not require an entrance examination.

### Measurement of blood pressure

BP was measured in the sitting position after 5 min of rest with the right arm held at heart level and by using an automated sphygmomanometer (Omron BP-203RVIII oscillometer; Nippon Colin, Tokyo, Japan). BP measurement was performed at 2 points: before 16 weeks and 20 weeks of gestation. If BP was measured several times before 16 weeks of gestation, the average systolic and diastolic value was calculated.

### Pregnancy-induced hypertension

PIH was defined according to the 2009 guideline for care and treatment of hypertension in pregnancy proposed by the Japanese Society of the Study of Hypertension in Pregnancy as “hypertension with or without proteinuria occurring after 20 weeks of gestation but resolving by 12 weeks postpartum” [20].

### Mediators and confounders

We considered the following potential mediators: pre-pregnancy BMI, smoking, family income, alcohol and salt consumption during pregnancy, and body weight gain until 20 weeks of gestation. The pre-pregnancy BMI was calculated as weight [kg]/height^2^ [m], which was obtained from the questionnaire at the time of enrollment. Data on maternal smoking and family income were also obtained through the questionnaire. Maternal salt and alcohol consumption during pregnancy was based on data obtained during pregnancy from a brief-type self-administered diet history questionnaire which had been used in a previous study [21].

Maternal age, parity, gestational age at BP measurement, pre-pregnancy complications (diabetes mellitus [DM], hypertension, and renal disease), previous pregnancy history of PIH, and family history of hypertension were used as potential confounders in this study. Information on maternal age and family history of hypertension was collected from the questionnaire. Data on parity, maternal pre-pregnancy complications, and previous history of PIH were obtained from medical charts and delivery records.

### Statistical analysis

Associations between the participants’ baseline information and educational levels were assessed by using ANOVA for continuous variables and *χ*^2^ or Fisher’s exact test for discrete variables. The association between educational levels and BP was assessed by multiple regression analysis applying the following models. First, we examined the association between BP and educational level in an unadjusted model. Second, confounders (i.e., gestational age at BP measurement, maternal age, parity, maternal pre-pregnancy complication of hypertension, DM, renal disease, previous pregnancy history of PIH, and family history of hypertension) were adjusted as basic model. Then, potential mediators (i.e., pre-pregnancy BMI, smoking, salt intake, alcohol intake, income, and body weight gain until mid-pregnancy) were added to the basic model one by one as covariates. Finally, we used a full model adjusted for all possible mediators and confounders. Similar to BP difference, the association between educational levels and PIH was assessed by multiple logistic regression analysis. A *P* value <0.05 was considered as statistically significant. All analyses were performed with the STATA software (Version 11.1 for Windows, USA).

## Results

Maternal characteristics of the study population stratified by educational level are shown in Table 1. Among the 923 participants, the number of participants in the high educational group was highest (n = 467, 50.6%), followed by the mid (n = 228, 24.7%) and low (n = 228, 24.7%) educational groups, which was skewed to higher educational attainment compared with mothers in the general population who delivered babies in Japan [22]. The mean age of participants was 33.7 years (standard deviation [SD] = 7.1). The participants in the low educational group were more likely to be overweight, parous, smokers, and to gain body weight until mid-pregnancy. Participants in the high educational group were more likely to have a larger annual household income. History of hypertension was most often seen in the mid educational group, probably by chance.

**Table 1:** Characteristics of sample stratified by educational level

**Characteristics**	**All (n= 923)**	Low (n=228)^*^	Mid (n=228)^*^	High (n=467)^*^	***P *value**
Maternal age (yrs)	33.7 (4.1)	33.4 (4.7)	34.1 (4.1)	33.6 (3.8)	NS
Pre-pregnancy BMI (kg/m^2^)	20.2 (2.3)	21.0 (2.9)	20.1 (2.2)	19.8 (2.0)	<0.0001
BMI>25, n(%)	41 (4.4)	19 (8.3)	10 (4.4)	12 (2.6)	<0.05
BMI>30, n(%)	3 (0.3)	2 (0.9)	1 (0.4)	0 (0)	NS
Parity					
0, n(%)	452 (49.0)	97 (42.5)	104 (45.6)	251 (53.8)	<0.0001
≧1, n(%)	471 (51.0)	131 (57.5)	124 (54.4)	216 (46.2)	
Mean gestational age before 16 weeks blood pressure	14.3 (0.98)	14.3 (0.98)	14.3 (0.98)	14.3 (1.0)	NS
Mean gestational age at 20 weeks blood pressure	20 (1.2)	19.9 (1.2)	20.1 (1.2)	20.0 (1.1)	NS
Maternal pre-pregnancy complications					
Diabetes mellitus, n(%)	5 (0.5)	3 (1.3)	1 (0.44)	1 (0.21)	NS
Hypertension, n(%)	5 (0.5)	0 (0.0)	4 (1.8)	1 (0.21)	<0.05
Renal disease, n(%)	4 (0.4)	1 (0.4)	1 (0.44)	2 (0.43)	NS
Pre-pregnancy complications					
PIH, n(%)	11 (1.2)	4 (1.8)	1 (0.44)	6 (1.3)	NS
Family History					
Diabetes mellitus, n(%)	72 (7.8)	14 (6.1)	19 (8.3)	39 (8.4)	NS
Hypertension, n(%)	69 (7.5)	13 (5.7)	14 (6.1)	42 (9.0)	NS
Smoking					
Never or former, n(%)	891 (96.9)	209 (92.5)	224 (98.7)	458 (98.1)	<0.001
Current, n(%)	29 (3.2)	17 (7.5)	3 (1.3)	9 (1.9)	
Income (per year), n(%)					
<4 million yen	49 (5.7)	23 (10.6)	10 (4.8)	16 (3.7)	<0.001
<6 million yen	199 (23.2)	78 (35.9)	50 (24.2)	71 (16.4)	
<8 million yen	189 (22.1)	56 (25.8)	47 (22.7)	86 (19.9)	
<10 million yen	185 (21.6)	34 (15.7)	44 (21.3)	107 (24.7)	
over 10 million yen	235 (27.4)	26 (12.0)	56 (27.1)	153 (35.3)	
Salt Intake					
low, n(%)	302 (34.1)	81 (37.7)	66 (30.0)	155 (34.3)	NS
moderate, n(%)	295 (33.3)	65 (30.2)	72 (32.7)	158 (35.0)	
high, n(%)	290 (32.7)	69 (32.1)	82 (37.3)	139 (30.8)	
Alcohol Intake					
None or former, n(%)	749 (84.4)	183 (85.1)	192 (87.3)	374 (82.7)	NS
Current, n(%)	138 (15.6)	32 (14.9)	28 (12.7)	78 (17.3)	
Body weight gain until midpregnancy (kg)	3.4 (2.5)	3.9 (2.9)	2.9 (2.6)	3.4 (2.2)	<0.001

Values are given as mean ± standard deviation for continuous variables.

PIH, pregnancy-induced hypertension; BMI, body mass index; NS, not significant; SD, standard deviation.

^*^”Low” denotes vocational training school, high school, or less; “Mid” denotes junior college; “High” denotes college or more than college.

**Figure 1:**
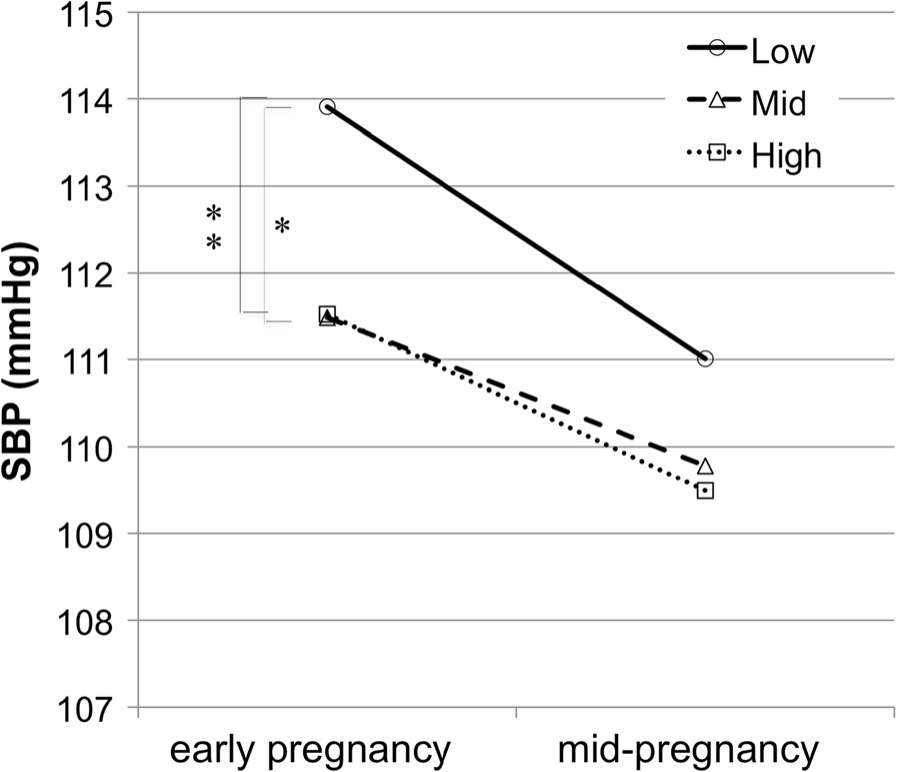
**Mean systolic blood pressure in early, mid-pregnancy stratified by educational level.** Mean blood pressure was significantly different from that in subgroup of women with low (*) and mid (**) educational level (P<0.05).

The systolic BP patterns from early to mid-pregnancy in each educational group are shown in Figure 1. Overall, the low educational group showed higher systolic values both in early and mid-pregnancy compared to the high and mid educational groups. For the systolic BP at early pregnancy, the BP value of the low educational group was significantly higher than that of the high (difference = 2.39 mmHg, 95% confidence interval [CI]: 0.59 to 4.19) and mid (difference = 2.43 mmHg, 95% CI: 0.34 to 4.52) educational groups. Similarly, the systolic BP at mid-pregnancy was higher in the low educational group than in the high (difference = 1.52 mmHg, 95% CI: –0.27 to 3.30) or mid educational groups (difference = 1.23 mmHg, 95% CI: –0.84 to 3.30). However, these differences were not significant.

**Figure 2:**
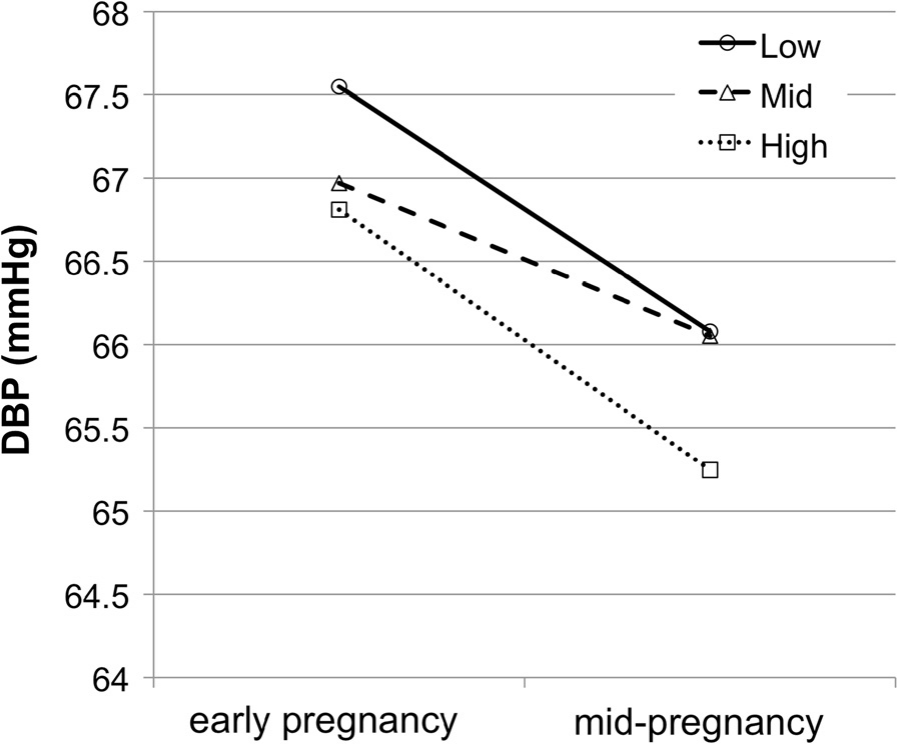
**Mean diastolic blood pressure in early, mid-pregnancy stratified by educational level.** Mean blood pressure was not significantly different from that in subgroup of women with low and mid educational level (P<0.05).

The diastolic BP patterns from early to mid-pregnancy in each educational group are shown in Figure 2. The mean diastolic BP in early pregnancy was higher in the group of low educational level than of high level (low vs. high, difference = 0.74 mmHg, 95% CI: –0.52 to 1.99; low vs. mid, difference = 0.58 mmHg, 95% CI: –0.88 to 2.03). However, these differences were not significant. Similarly, no statistical differences between the educational groups were observed in mid-pregnancy. Decreases in both systolic and diastolic BP from early to mid-pregnancy were observed in all educational groups.

The associations between systolic and diastolic BP at early pregnancy and educational levels are shown in Table 2. In the basic model adjusted for maternal age, parity, gestational age at BP measurement, pre-pregnancy maternal complications, including DM, hypertension, and renal disease, previous pregnancy history of PIH, and family history of hypertension, the mean systolic BP difference between low and high educational groups remained significant (difference = 2.42 mmHg, 95% CI: 0.61 to 4.23). Furthermore, after adding pre-pregnancy BMI to the basic model, the difference in systolic BP between low and high educational groups became no longer statistically significant (difference = 1.19 mmHg, 95% CI: –0.62 to 3.00). Adding other possible mediators (i.e., smoking, salt and alcohol intake, weight gain until mid-pregnancy) did not further attenuate the difference in BP between the low vs. high educational groups, with the exception of income. When the pre-pregnancy BMI was added in the full model, BP was not statistically different between the educational groups. With regard to the diastolic BP, the BP differences between the educational groups were not statistically significant. However, similar to systolic BP, diastolic BP differences were attenuated after adding pre-pregnancy BMI to the basic model.

**Table 2:** The associations between systolic and diastolic blood pressure at early pregnancy and educational level (and 95% CIs) (n=923)^*^

**Educational level**	**Unadjusted model**	**Basic model†**	**Basic model† +BMI**	**Basic model† +smoking**	**Basic model† +salt intake**	**Basic model† +alcohol intake**	**Basic model† +income**	**Basic model† +BW gain**	**Full model‡**
**SBP, mmHg**									
High	Reference	Reference	Reference	Reference	Reference	Reference	Reference	Reference	Reference
Mid	−0.04 (−1.84 to 1.76)	−0.28 (−2.08 to 1.52)	−0.46 (−2.23, 1.30)	−0.15 (−1.96, 1.67)	−0.41 (−2.25, 1.42)	−0.49 (−2.32, 1.34)	−0.81 (−2.69, 1.06)	−0.32 (−2.15, 1.51)	−1.06 (−2.96, 0.84)
Low	2.39 (0.59 to 4.19)	2.42 (0.61 to 4.23)	1.19 (−0.62, 3.00)	2.85 (0.94, 4.76)	2.37 (0.52, 4.23)	2.23 (0.38, 4.08)	1.46 (−0.47, 3.38)	2.21 (0.36, 4.06)	0.13 (−1.94, 2.20)
**DBP, mmHg**									
High	Reference	Reference	Reference	Reference	Reference	Reference	Reference	Reference	Reference
Mid	0.16 (−1.09 to 1.41)	−0.05 (−1.29 to 1.20)	−0.16 (−1.38, 1.06)	0.13 (−1.12, 1.38)	−0.23 (−1.49, 1.03)	−0.32 (−1.58, 0.93)	−0.088 (−1.39, 1.21)	−0.10 (−1.37, 1.16)	−0.29 (−1.60, 1.03)
Low	0.74 (−0.52 to 1.99)	0.79 (−0.46 to 2.04)	0.041 (−1.21, 1.30)	1.31 (−0.0043, 2.63)	0.62 (−0.66, 1.90)	0.56 (−0.71, 1.83)	0.42 (−0.92, 1.75)	0.75 (−0.53, 2.02)	−0.26 (−1.69, 1.17)

^*^ Estimates denote difference in BP (mmHg) between low/mid and high educational level.

†Basic model: adjusted for gestational age at BP measurement, maternal age, parity, past medical history of hypertension, pregnancy-induced hypertension, diabetes mellitus, renal disease, family history of hypertension.

‡Full model: Basic model+ pre-pregnancy BMI, smoking, salt intake, alcohol intake, income, BW gain until mid-pregnancy.

Abbreviations: SBP, systolic blood pressure; DBP, diastolic blood pressure, BMI, pre-pregnancy body mass index; BW gain, body weight gain until mid-pregnancy.

The associations between systolic and diastolic BP at mid-pregnancy and educational level are shown in Table 3. In the basic model, the BP in the low educational group was significantly higher than that in the high educational group (difference = 1.84 mmHg, 95% CI: 0.057 to 3.61). Similar to the results shown in Table 2, after adding pre-pregnancy BMI to the basic model, BP differences became smaller and were not anymore statistically significant. Interestingly, adding smoking to the basic model increased the BP difference which resulted in statistical significance (difference = 2.53 mmHg, 95% CI: 0.66 to 4.40). With regard to the diastolic BP, educational levels were not statistically associated. Similar to early pregnancy, adding BMI to the basic model attenuated the differences in diastolic BP.

**Table 3:** The associations between systolic and diastolic blood pressure at mid-pregnancy and educational level (and 95% CIs) (n=923)^*^

**Educational level**	**Unadjusted model**	**Basic model†**	**Basic model† +BMI**	**Basic model† +smoking**	**Basic model† +salt intake**	**Basic model† +alcohol intake**	**Basic model† +income**	**Basic model† +BW gain**	**Full model‡**
**SBP, mmHg**									
High	Reference	Reference	Reference	Reference	Reference	Reference	Reference	Reference	Reference
Mid	0.29 (−1.50, 2.07)	−0.0063 (−1.77, 1.76)	−0.20 (−1.92, 1.52)	0.17 (−1.60, 1.95)	−0.14 (−1.94, 1.66)	−0.18 (−1.98, 1.62)	−0.31 (−2.16, 1.54)	0.32 (−1.46, 2.10)	0.085 (−1.75, 1.92)
Low	1.52 (−0.27, 3.30)	1.84 (0.057, 3.61)	0.58 (−1.20, 2.35)	2.53 (0.66 to 4.40)	1.87 (0.045, 3.69)	1.76 (−0.057, 3.59)	1.17 (−0.73, 3.06)	1.77 (−0.035, 3.57)	0.53 (−1.48, 2.53)
**DBP, mmHg**									
High	Reference	Reference	Reference	Reference	Reference	Reference	Reference	Reference	Reference
Mid	0.81 (−0.42, 2.03)	0.58 (−0.62, 1.78)	0.47 (−0.71, 1.66)	0.73 (−0.48, 1.94)	0.40 (−0.82, 1.62)	0.37 (−0.86, 1.59)	0.14 (−1.10, 1.37)	0.69 (−0.53, 1.90)	0.17 (−1.08, 1.41)
Low	0.83 (−0.39, 2.06)	0.99 (−0.22 to 2.20)	0.30 (−0.92, 1.52)	1.44 (0.17 to 2.72)	1.10 (−0.13 to 2.34)	1.02 (−0.22, 2.25)	0.42 (−0.85, 1.69)	1.00 (−0.23, 2.22)	0.06 (−1.31, 1.42)

^*^ Estimates denote difference in BP (mmHg) between low, mid and high educational level.

†Basic model: adjusted for gestational age at BP measurement, maternal age, parity, past medical history of hypertension, pregnancy-induced hypertension, diabetes mellitus, renal disease, family history of hypertension.

‡Full model: Basic model+ pre-pregnancy BMI, smoking, salt intake, alcohol intake, income, BW gain until mid-pregnancy.

Abbreviations: SBP, systolic blood pressure; DBP, diastolic blood pressure, BMI, pre-pregnancy body mass index; BW gain, body weight gain until mid-pregnancy.

The odds ratio (OR) of PIH by educational level is shown in Table 4. In total, PIH was diagnosed in 23 cases (2.5%). In the unadjusted model, the point estimate of the OR of PIH of the low educational group was 1.24 (95% CI: 0.44 to 3.44) in comparison with high educational group. In the full model adjusted for all confounders and mediators, the point estimate of the OR of PIH of the low educational group was 1.19 (95% CI: 0.31 to 4.60) in comparison with the high educational group, suggesting that the low educational group was 1.19 times more likely to develop PIH than the high educational group, even though the OR was not statistically significant.

**Table 4:** Odds ratio (and 95% confidence intervals) of pregnancy-induced hypertension stratified by educational level (n=923)

**Educational level**	**N (%)**	**Unadjusted**	**Full model‡**
**PIH**	23 (2.5)		
High (n=467)	10 (2.1)	Reference	Reference
Mid (n=228)	7 (3.1)	1.45 (0.54, 3.85)	0.44 (0.076, 2.60)
Low (n=228)	6 (2.6)	1.24 (0.44, 3.44)	1.19 (0.31, 4.60)

‡Full model: adjusted for maternal age, parity, past medical history of hypertension, pregnancy-induced hypertension.

Diabetes mellitus, renal disease, Family history of hypertension, pre-pregnancy body mass index, smoking, Salt intake.

Alcohol intake, income, body weight gain until mid-pregnancy.

## Discussion

In our study, we found that among Japanese pregnant women the low educational group had significantly higher systolic BP than the high educational groups in early pregnancy and that this group maintained higher systolic and diastolic BPs toward mid-pregnancy. Furthermore, we found that the pre-pregnancy BMI mediated the association between educational levels and BP. On the other hand, smoking, alcohol intake, salt intake, and body weight gain until mid-pregnancy did not mediate this association.

The results of the current study are consistent with those of a previous study on SES and BP during pregnancy [13]. Silva et al. reported that the low educational group had significantly higher BPs throughout pregnancy. They categorized the educational level into 4 levels: high (university or higher), mid-high (higher vocational training), mid-low (less than 3 years of general secondary school, intermediate vocational training completed, or first year of higher vocational training), and low (no education, primary school, lower vocational training, intermediate general school, or ≤3 years of general secondary school). The systolic and diastolic BP difference between the low and high educational groups was 2.67 mmHg (95% CI: 0.66 to 4.40) and 0.53 mmHg (95% CI: –0.58 to 1.64), respectively, at early pregnancy (less than 18 weeks of gestation). Even though the categorization of educational level in our study is different from their study, our results are similar; in our study, the systolic and diastolic BP difference between the low and high educational groups was 2.39 mmHg (95% CI: 0.59 to 4.19) and 0.74 mmHg (95% CI: –0.52 to 1.99), respectively, at early pregnancy.

A decrease in systolic and diastolic BP from early to mid-pregnancy, known as a protective factor for PIH [13,23], was observed among every educational group in our study. In Silva’s study, no decrease in diastolic BP from early to mid-pregnancy was reported among the low educational group. This inconsistency of BP decrease by educational groups might be due to different characteristics of the study population. Compared with Silva’s study, our sample population was less likely to be overweight (pre-pregnancy BMI; this study vs. Silva’s study, 20.2 ± 2.3 vs. 23.2 ± 3.9) and to consume alcohol during pregnancy (this study vs. Silva’s study, 15.6% vs. 47.1%). Furthermore, differences in the health care system for pregnant women might contribute to these differences, i.e., prenatal care in Japan might be associated with BP control, and quick access to a hospital with universal health insurance might be considered to have a beneficial effect on BP control.

Regarding mediators, the same previous study reported that the pre-pregnancy BMI mediates the association between educational level and BP [13], whereas smoking, alcohol intake, and body weight gain until mid-pregnancy do not mediate this association. Our results confirmed the results of the previous study and added to the literature that salt intake do not mediate the association between educational levels and BP during pregnancy.

From the results of our study, smoking showed an inverse association with BP, i.e., smoking pregnant women showed lower systolic and diastolic BP. Yet, smoking mediates, in part, the association between SES and cardiovascular events in adults [24]. Habitual smokers generally have a lower BP than non-smokers [25], which is related to lower body weight [26]. In pregnant women, it is reported that smoking is associated with lower diastolic BP until mid-pregnancy compared with non-smoking women [27]. Our results are also in line with those results. Smoking was associated with lower systolic and diastolic BP until mid-pregnancy, and this association did not change among the different educational groups.

Although not statistically significant, the point estimate of the OR for PIH in the low educational group was 1.19 compared with the high educational group, suggesting that the educational level might be associated with PIH. The non-significant result could be due to small sample size, i.e., the number of PIH cases was small in our study. Previous reports on the association between low educational level and PIH are inconsistent. On the basis of results of a population-based, prospective cohort study, Silva et al. reported that low educational level was significantly associated with the occurrence of preeclampsia [28] and gestational hypertension [29]. Haelterman et al. conducted a case–control study of 99 severe preeclampsia cases and reported that individuals with a low educational level (primary school or below) had a statistically higher OR (OR = 2.3, 95% CI: 1.2 to 4.4) than those who attended a primary school or higher [30]. On the other hand, by using birth records from North Carolina, Savitz et al. reported that maternal education did not differ with regard to PIH occurrence, although the process of diagnosing PIH might be potentially fallible with possible underascertainment [31]. In our study, the diagnosis of PIH is considered accurate because it was made by a single obstetrician (S.C.J.) on the basis of the same criteria. Further research is necessary to confirm the association between maternal educational level and PIH using a larger sample size.

There are several limitations in our study. First, this study was conducted retrospectively at a single center with a relatively small sample size. Because of the small number of participants with PIH, we were not able to separate PIH into true preeclampsia (hypertension with proteinuria). To analyze the subtype, a study with a larger sample size should be performed. Second, the percentage of participants who graduated from high school or less was also relatively small and the proportion of the high educational group was high in comparison with the general population among those who delivered babies in Japan [22] suggesting sampling bias. However, we demonstrated an association between educational level and BP even among higher SES pregnant mothers. Third, unmeasured possible mediators and confounders, e.g., maternal birth weight [32], exercise during pregnancy [33], and neighborhood effects [34], might exist. Fourth, since some of the participants were recruited beyond the first trimester, and pre-pregnancy body weight and height were self-reported by questionnaire, pre-pregnancy BMI might be underestimated. Thus, a further study that replicates these study findings using a population-based, multicenter, large prospective cohort study is essential.

On the basis of our study results, a policy on body weight control may be effective targeting people who do not attend junior college or university in order to prevent higher BP during pregnancy. In the United State, where child and adolescent obesity is a growing problem, health education on body weight control is implemented among high school students aiming for future BP control [35]. A similar health policy might be effective in Japan.

## Conclusion

In conclusion, among Japanese pregnant women, the low educational group had significantly higher systolic BP than the mid and high educational groups in early pregnancy and mid-pregnancy. The pre-pregnancy BMI mediated the association between educational levels and BP. Thus, education on body weight control in high schools might be useful to control BP during pregnancy, which would prevent the onset of PIH.

## Abbreviations

BP: Blood pressure; BMI: Body mass index; CI: Confidence interval; DM: diabetes mellitus; OR: Odds ratio; PIH: Pregnancy induced hypertension; SD: Standard deviation; SES: Socioeconomic status

## Competing interests

The authors declared no competing interest.

## Authors’ contributions

SCJ analyzed data and wrote first draft of manuscript. TF conceived design, interpreted the results, and finalized the manuscript. AH and HS contributed interpretation of data. NA and YO contributed acquisition of data. All authors read and approved the final manuscript.

## Pre-publication history

The pre-publication history for this paper can be accessed here:

http://www.biomedcentral.com/1471-2458/13/389/prepub
